# Elevated Soluble TNF-Receptor 1 in the Serum of Predementia Subjects with Cerebral Small Vessel Disease

**DOI:** 10.3390/biom13030525

**Published:** 2023-03-13

**Authors:** Kaung H. T. Salai, Liu-Yun Wu, Joyce R. Chong, Yuek Ling Chai, Bibek Gyanwali, Caroline Robert, Saima Hilal, Narayanaswamy Venketasubramanian, Gavin S. Dawe, Christopher P. Chen, Mitchell K. P. Lai

**Affiliations:** 1 Department of Pharmacology, Yong Loo Lin School of Medicine, National University of Singapore, Singapore 117600, Singapore; 2 Healthy Longevity Translational Research Programme, Yong Loo Lin School of Medicine, National University of Singapore, Singapore 117456, Singapore; 3 Memory Aging and Cognition Centre, National University Health System, Singapore 117600, Singapore; 4 Saw Swee Hock School of Public Health, National University of Singapore, Singapore 117597, Singapore; 5 Departments of Epidemiology and Radiology & Nuclear Medicine, Erasmus University Medical Center, 3015 GD Rotterdam, The Netherlands; 6 Raffles Neuroscience Centre, Raffles Hospital, Singapore 188770, Singapore; 7 Precision Medicine Translational Research Programme, Yong Loo Lin School of Medicine, National University of Singapore, Singapore 117596, Singapore; 8 Neurobiology Programme, Life Sciences Institute, Centre for Life Sciences, National University of Singapore, Singapore 117456, Singapore

**Keywords:** Alzheimer’s disease, biomarker, cerebral small vessel diseases, predementia, serum, TNF-receptor 1, tumor necrosis factor, vascular cognitive impairment

## Abstract

Tumor necrosis factor-receptor 1 (TNF-R1)-mediated signaling is critical to the regulation of inflammatory responses. TNF-R1 can be proteolytically released into systemic blood circulation in a soluble form (sTNF-R1), where it binds to circulating TNF and functions to attenuate TNF-mediated inflammation. Increases of peripheral sTNF-R1 have been reported in both Alzheimer’s disease (AD) dementia and vascular dementia (VaD). However, the status of sTNF-R1 in predementia subjects (cognitive impairment, no dementia, CIND) is unknown, and putative associations with cerebral small vessel disease (CSVD), as well as with longitudinal changes in cognitive functions are unclear. We measured baseline serum sTNF-R1 in a longitudinally assessed cohort of 93 controls and 103 CIND, along with neuropsychological evaluations and neuroimaging assessments. Serum sTNF-R1 levels were increased in CIND compared with controls (*p* < 0.001). Higher baseline sTNF-R1 levels were specifically associated with lacunar infarcts (rate ratio = 6.91, 95% CI 3.19–14.96, *p* < 0.001), as well as lower rates of cognitive decline in the CIND subgroup. Our data suggest that sTNF-R1 interacts with vascular cognitive impairment in a complex manner at predementia stages, with elevated levels associated with more severe CSVD at baseline, but which may subsequently be protective against cognitive decline.

## 1. Introduction

Alzheimer’s disease (AD) and vascular dementia (VaD) are the two most common forms of dementia [1]. AD is a neurodegenerative disease characterized by the accumulation of extracellular amyloid plaques and intracellular neurofibrillary tangles [1]. In contrast, VaD falls within the vascular cognitive impairment (VCI) spectrum and is associated with cerebrovascular disease, particularly cerebral small vessel disease (CSVD) [2]. CSVDs manifest on magnetic resonance imaging (MRI) scans as lacunes, white matter hyperintensities (WMHs) and cerebral microbleeds (CMBs) [2]. The presence of CSVD has been associated with cognitive impairment and an increased risk of dementia [2], and also acts additively or synergistically with AD pathology [3]. Therefore, in certain populations where the baseline CSVD burden is relatively high, such as those in Asia, the cerebrovascular contribution to cognitive decline and dementia may be more prominent than in Western populations [4,5], and more research efforts should be expended on uncovering the common pathophysiological mechanisms underlying CSVD and AD, as well as identifying potential biomarkers and therapeutic targets. 

One pathogenic factor observed in both CSVD and AD is dysregulated neuroinflammation, characterized by chronic gliosis within compromised brain regions [6,7]. In AD, a proinflammatory state with widespread upregulation of cytokines and chemokines has been observed, and neuroinflammation is also implicated in the pathogenesis of amyloid plaques and neurofibrillary tangles [8]. In patients with CSVD, microglia and peripheral macrophages may play both protective and detrimental roles in maintaining cerebral vasculature integrity [6]. Furthermore, intracerebral ventricular administration of tumor necrosis factor (TNF), an acute-phase proinflammatory cytokine known to be secreted by both microglia and macrophages [9], dose-dependently increased infarct volume in rodent models of brain ischemia [10]. Studies also showed increased cerebrospinal fluid (CSF) levels of TNF in patients with VaD [11]. Interestingly, the high-affinity subtype of the receptor for TNF, TNF-receptor 1, can be cleaved from its membrane-bound state and secreted into the systemic blood circulation in a soluble form (sTNF-R1) as a response to various upstream stimuli, including TNF [12,13]. The release of sTNF-R1 may in turn attenuate TNF-induced inflammatory processes by acting as decoy receptors for circulating TNF [12]. As blood sTNF-R1 is detectable for prolonged periods, it has been proposed to be a more reliable indicator of circulating TNF concentrations than TNF itself [12], suggesting potential utility as a promising inflammatory biomarker for individuals with CSVD and dementia.

In AD and VaD subjects, plasma sTNF-R1 has previously been reported to be increased compared with healthy controls [14,15]. In patients with pre-clinical stages of dementia, CSF sTNF-R1 was positively correlated with CSF β-amyloid and tau [16]. However, while increased plasma levels of sTNF-R1 were associated with memory functions in healthy individuals [17], the putative links between sTNF-R1 and cognitive performance, as well as structural brain changes in predementia subjects, remain unclear. Within the spectrum of cognitive impairment and dementia, predementia (which may include MCI or cognitive impairment, no dementia (CIND)) represents a clinically important stage when disease-modifying interventions may still be possible [18,19]. Furthermore, associations between sTNF-R1 and neuroimaging features of CSVD await investigation. Therefore, we aimed to examine serum sTNF-R1 associations in a prospectively assessed cohort of predementia subjects with neuroimaging markers of CSVD and cerebral atrophy at baseline, as well as the relationship between sTNF-R1 and longitudinal changes in cognitive function in the cohort of patients with CIND over 3 years of follow-up.

## 2. Materials and Methods

### 2.1. Study Cohort

The selection and assessment of subjects for this study have been previously described [20]. Briefly, patients with subjective complaints of memory loss were recruited from memory clinics at Singapore’s National University Hospital and Saint Luke’s Hospital. Subjects underwent clinical, physical and neuropsychological assessments, together with neuroimaging at the National University of Singapore. Important demographic and medical covariates, including cardiovascular risk factors (see Section 2.4 on covariates below) and exclusion factors (previous head trauma, psychiatric illnesses, thyroid disease and non-dementia neurodegenerative conditions), were collected by detailed questionnaires and reviews of medical records. The inclusion criteria of this study were as follows: (i) patients diagnosed as no cognitive impairment (NCI, used as control group) and CIND at baseline, (ii) complete neuropsychological data at baseline and ≥2 follow-up visits and (iii) sufficient baseline serum available for sTNF-R1 measurement. The diagnoses of all study subjects were made at regular consensus meetings attended by study clinicians and neuropsychologists. NCI was defined as those found to be cognitively normal and without functional loss based on the standard neuropsychological test battery (see below). CIND cases were defined as not meeting the Diagnostic and Statistical Manual of Mental Disorders, Fourth Edition (DSM-IV) diagnostic criteria for dementia but showing impairment in at least one domain of the neuropsychological battery (that is, education-adjusted cognitive scores ≥1.5 standard deviations (SDs) below cut-off scores on any test) [21]. As this study is focused on predementia, patients diagnosed with AD dementia or VaD based on current criteria [22,23] were excluded.

### 2.2. Standard Protocol Approvals, Registrations and Patient Consent

This study was performed in accordance with the Declaration of Helsinki and approved by the Singapore National Healthcare Group Domain-Specific Review Board (NHG-DSRB) (reference: 2010/00017; study protocol number: DEM4233). Written informed consent was obtained from all participants or their caregivers.

### 2.3. Neuropsychological Battery

All patients were administered an annual comprehensive neuropsychological battery which consists of six cognitive domains (see Supplementary Table S1 for the component tests of each domain) [24]. Raw scores of individual tests were transformed into standardized *z*-scores using the mean and standard deviation (SD) of the control group. The score for each cognitive domain was created by averaging the *z*-scores of individual component tests and standardized using the composite mean and SD of the NCI. In order to obtain the global cognitive score for each patient, the domain *z*-scores were averaged and standardized using the mean and SD of the NCI group, as previously described [25]. The follow-up annual visits’ global and domain-specific cognitive *z*-scores were obtained using the means and SDs of the NCI group at baseline. 

### 2.4. Covariates

Together with the demographic variables, vascular risk factors (hypertension, hyperlipidemia, diabetes mellitus and cardiovascular disease) were collected and classified as absent or present. Hypertension was determined by a systolic blood pressure ≥140 mmHg and/or diastolic blood pressure ≥90 mmHg, or the patient being on medications for hypertension. Hyperlipidemia was determined by total cholesterol levels of ≥4.14 mM, or the patient being on medications for hyperlipidemia. Diabetes mellitus was defined by glycated hemoglobin (HbA1c) of ≥6.5%, or the patient being on medications for diabetes mellitus. Medications for hypertension, hyperlipidemia and diabetes mellitus were also recorded. Cardiovascular disease was determined by a previous history of atrial fibrillation, congestive heart failure or myocardial infarction. Apolipoprotein E (APOE) genotyping was performed as previously described [26] to determine positive APOE ε4 carrier status (presence of at least one APOE ε4 allele). 

### 2.5. Neuroimaging

Using a 3T Siemens Magnetom Trio Tim scanner with a 32-channel head coil, MRI scans were obtained at the Clinical Imaging Research Center of the National University of Singapore. For each subject, the following MRI markers were determined as previously described [27,28]. Lacunar infarcts (lacunes) were identified as lesions involving the subcortical regions, 3–15 mm in diameter, with a low signal on the T1-weighted image and a fluid attenuated inversion recovery (FLAIR) sequence giving a high signal on the T2-weighted image, as well as a hyperintense rim with a center following CSF intensity on FLAIR. WMHs were identified as hyperintense regions on T2 and FLAIR sequences without cavitation and hypointense on T1-weighted images. WMHs were graded using the Age-Related White Matter Changes (ARWMC) scale [29]. Cerebral microbleeds (CMBs) were identified as small focal rounded hypointense lesions, graded based on susceptibility-weighted imaging sequences using the Brain Observer MicroBleed Scale [30]. Medial temporal lobe atrophy (MTA) was graded by coronal sections on a 5-point Scheltens’ scale (0—normal, 1—mild, 2—mild to moderate, 3—moderate, 4—severe), which considers the widening of the choroid fissure and temporal horn and loss of hippocampal height [28]. The presence vs. absence of significant neurodegeneration predictive of AD [31] was defined by MTA scores of 2–4 vs. scores of 0–1. Furthermore, the presence vs. absence of significant CSVD burden was defined as: confluent WMHs (ARWMC score ≥ 8 vs. <8), ≥2 lacunes vs. <2 lacune or ≥2 CMBs vs. <2 CMBs.

### 2.6. Serum sTNF-R1 Measurements

Non-fasting blood was drawn from patients into serum-separating tubes; centrifuged at 2000× *g* for 10 min at 4 °C. Serum samples were mixed well, aliquoted, and stored at −80 °C for future use. All samples underwent only one freeze–thaw cycle. Samples were diluted by 10× volume with the kit’s diluent, then assayed for sTNF-R1 concentrations by quantitative sandwich enzyme immunoassay technique (Quantikine^®^ ELISA Kit, R&D Systems Inc., Minneapolis, MN, USA), as per the manufacturer’s instructions. sTNF-R1 was detectable and linear from 7.8 pg/mL to 500 pg/mL.

### 2.7. Statistical Analyses

The baseline serum biomarker (sTNF-R1) level and clinical data were used for cross-sectional analyses, while the global and cognitive domain-specific *z*-scores data were obtained at baseline, as well as up to three years follow-up (mean 2.9 ± SD 0.3 years) for longitudinal analyses of cognitive trajectories. Analyses of data were performed using the SPSS (version 26, IBM Inc., Armonk, NY, USA) and R (version 4.0.5, R Foundation) software. Independent samples t-tests, Mann–Whitney U tests or chi-square tests were used to compare demographic characteristics and sTNF-R1 levels between NCI and CIND. For regression analyses, sTNF-R1 levels were stratified into tertiles (lowest tertile: ≤1054.04 pg/mL, middle tertile: 1054.05–1333.69 pg/mL, highest tertile: ≥1333.70 pg/mL) as sTNFR1 concentrations were not normally distributed in this study (Shapiro–Wilk test *p* < 0.001, skewness = 1.34, kurtosis = 2.36), then examined for associations with CSVD markers at baseline. Poisson regression modelling was performed for counts of lacunes, while negative binomial regression was used for over-dispersed CMB counts and expressed as rate ratios (RR) with 95% confidence intervals (95% CI). For WMH grading, linear regression models were performed with measures of associations by mean differences (MD) with 95% CI. Binary logistic regression was used for significant MTA and expressed as odds ratios (OR) with 95% CI. Models were then adjusted with various covariates (see Section 2.4. Covariates above). 

Lastly, we used linear mixed-effect models to examine the associations between baseline serum sTNF-R1 levels (independent variable) and changes in both global and domain-specific cognitive *z*-scores (dependent variables) of CIND subjects over time. All models included random effects for subjects and fixed effects for baseline levels of sTNF-R1 (lowest, middle and highest tertiles) and time, as well as interactions between baseline sTNF-R1 and time. Covariates added to the models included age, gender, education, APOE4 carrier status, hypertension, diabetes mellitus, cardiovascular disease, and hyperlipidemia. The model estimates were produced using the maximum likelihood method with random intercept and slope.

## 3. Results

### 3.1. Study Participants

Of the 273 participants diagnosed as NCI or CIND in the cohort, 186 subjects had available collected blood samples, as well as complete neuroimaging data at baseline (see Supplementary Figure S1). Similar to previous observations for our cohort [32,33,34,35], CIND subjects were significantly older (*p* < 0.001) with fewer years of education (*p* = 0.006) than NCI subjects. Therefore, both demographic variables (age and education) are included as covariates for subsequent regression analyses. Table 1 shows that sTNF-R1 levels in CIND were significantly higher than NCI (*p* < 0.001). 

### 3.2. Associations between sTNF-R1 and Neuroimaging Markers

**Figure 1:**
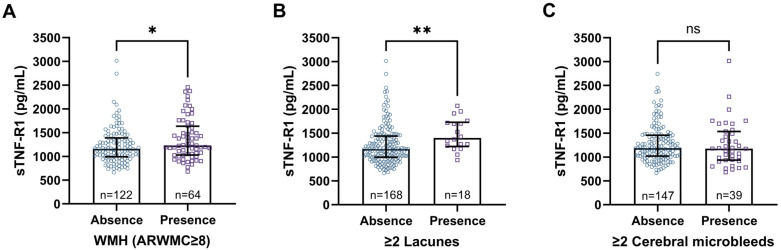
Serum sTNF-R1 concentrations in predementia subjects with CSVD. Bar graphs are median sTNF-R1 values ± interquartile range (IQR) stratified by the absence vs. presence of significant (**A**) WMH, (**B**) lacunes and (**C**) cerebral microbleeds (see Section 2.5 on neuroimaging for definitions). Datapoints represent available individual measures. * *p* < 0.05, ** *p* < 0.01, ns: not significant, pairwise comparisons using independent-samples Mann–Whitney U-tests. ARWMC = age-related white matter changes; *n* = number of cases; WMH = white matter hyperintensities.

In order to investigate potential associations between sTNF-R1 levels and CSVD, we compared sTNF-R1 levels between subjects with and without significant WMH, lacunes and cerebral microbleeds (total *n* = 186). sTNF-R1 concentrations were significantly higher in subjects with confluent WMH (ARWMC score ≥ 8, *p* = 0.034) or the presence of lacunes (*p* = 0.004) (Figure 1). No significant differences were observed in subjects with or without significant cerebral microbleeds (*p* = 0.621). In unadjusted univariate regression models on the entire cohort, higher sTNF-R1 (analyzed as tertiles) was significantly associated with the severity of WMH (as measured by ARWMC scores) and the number of lacunes and cerebral microbleeds (Table 2). After adjusting for covariates, sTNF-R1 remained significantly associated with lacunes but not with WMH and cerebral microbleeds (Table 2). Interestingly, sTNF-R1 was not associated with significant MTA after adjusting for covariates (Supplementary Table S2), suggesting that sTNF-R1 may be a biomarker of CSVD rather than the pattern of neurodegeneration which is characteristic of AD. 

### 3.3. Associations between sTNF-R1 and Cognitive Trajectories

**Figure 2:**
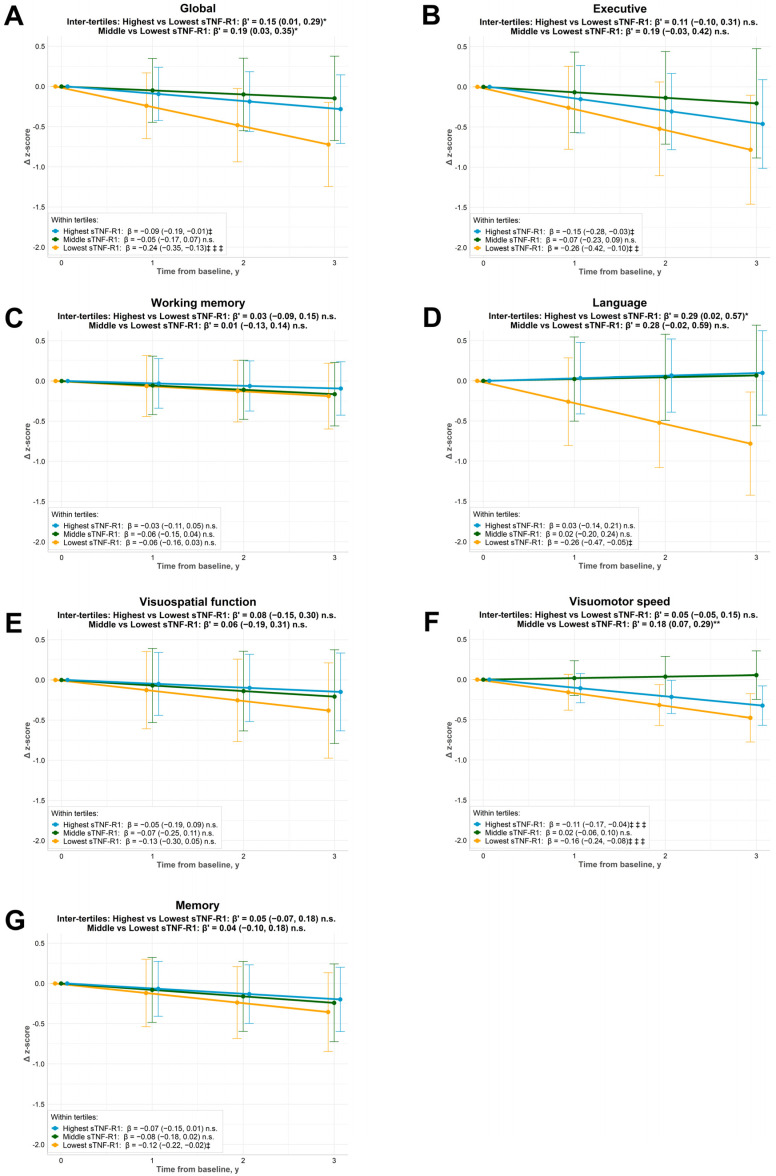
Associations between baseline sTNF-R1 tertiles and the cognitive trajectories of CIND subjects. Estimated mean change in cognitive domain scores (∆ *z*-scores) with 95% CI are stratified by the tertiles of sTNF-R1 (yellow: lowest tertile (*n* = 30), green: middle tertile (*n* = 27), blue: highest tertile (*n* = 46)) for (**A**) global cognition as well as (**B**–**G**) each of the six cognitive domains (see Section 2.3 on neuropsychological battery). β-coefficients with 95% CIs are derived from linear mixed-effect models adjusted for age, education, gender, APOE4 carrier status, hypertension, diabetes mellitus, cardiovascular disease and hyperlipidemia. Interpretation: Within tertiles, β represents the decline in cognitive *z*-scores over time for each tertile of sTNF-R1; ‡ *p* < 0.05, ‡‡ *p* < 0.01, ‡‡‡ *p* < 0.001, n.s.: not significant. Between tertiles, β’ represents the difference in the rate of change in cognitive *z*-scores between the highest/middle tertiles of sTNF-R1 vs. the lowest sTNF-R1 tertile; * *p* < 0.05, ** *p* < 0.01, n.s.: not significant. CIND = cognitive impairment, no dementia; *n* = number of cases.

Interestingly, while no significant difference in baseline cognitive *z*-scores was observed among the tertiles of sTNF-R1 (Supplementary Figure S2), we found that sTNF-R1 was associated with the cognitive trajectories of CIND subjects, in that significant declines in scores over time were observed within specific sTNF-R1 tertiles for global cognition (lowest: β = −0.24, 95% CI = −0.35 to −0.13; highest: β = −0.09, 95% CI = −0.19 to −0.01, Figure 2A), executive function (lowest: β = −0.26, 95% CI = −0.42 to −0.10; highest: β = −0.15, 95% CI = −0.28 to −0.03, Figure 2B), language (lowest: β = −0.26, 95% CI = −0.47 to −0.05, Figure 2D), visuomotor speed (lowest: β = −0.16, 95% CI = −0.24 to −0.08; highest: β = −0.11, 95% CI = −0.17 to −0.04, Figure 2F) and memory (lowest: β = −0.12, 95% CI = −0.22 to −0.02, Figure 2G). However, subjects with higher sTNF-R1 showed slower cognitive declines compared to those with the lowest sTNF-R1 tertile in global cognition (highest vs. lowest: β’ = 0.15, 95% CI = 0.01 to 0.29; middle vs. lowest: β’ = 0.19, 95% CI = 0.03 to 0.35, Figure 2A), language (highest vs. lowest: β’ = 0.29, 95% CI = 0.02 to 0.57, Figure 2D) and visuomotor speed (middle vs. lowest: β’ = 0.18, 95% CI = 0.07 to 0.29, Figure 2F).

## 4. Discussion

This study reports significantly higher levels of serum sTNF-R1 in CIND compared to NCI patients, and extends previous findings of elevated sTNF-R1 in dementia patients [14,15] to the predementia (also known as minimal cognitive impairment, MCI) stages. Furthermore, we investigated potential associations between sTNF-R1 and CSVD, showing that higher levels of sTNF-R1 were specifically linked to baseline measurements of lacunes independent of vascular risk factors. While the precise mechanisms linking sTNF-R1 with lacunes are unknown, postmortem and in vivo studies have reported the migration of microglia toward regions of infarcts after an ischemic episode, whereupon the secretion of chemoattractants, including TNF [9], initiates the recruitment of macrophages, monocytes and granulocytes, as well as the perpetuation of neuroinflammatory responses [36,37]. Interestingly, the production of TNF under chronic inflammatory conditions has been known to induce sTNF-R1 secretion into the systemic blood circulation [38], and data from mouse models suggest that sTNF-R1 upregulation binds to peripheral free TNF, thus attenuating TNF-mediated inflammatory responses and ameliorating increases in infarct size after middle cerebral artery occlusion [10,39]. Considering the current findings of sTNF-R1 in association with lacunes, we postulate that sTNF-R1 might be an adaptive response to the increased TNF and may play a protective role in TNF-associated CSVD processes, thus underscoring the biological relevance of sTNF-R1 as a biomarker for CSVD. Regarding lacunes, some studies have reported associations with worse cognitive outcomes [40], while others have not [41]. Nevertheless, successful validation of peripheral sTNF-R1 as a biomarker for the early identification of individuals with cerebral vascular pathologies may allow clinicians to prioritize timely management of vascular risk factors in these patients, thereby lowering the incidence of VCI, and potentially preventing concomitant AD progression [42]. Our data may have added clinical significance for Asian populations which are known to have a relatively high CSVD burden [3,4]. Moreover, compared with expensive MRI scans, peripheral biomarkers may be a cost-effective alternative for at-risk populations, especially those from developing countries [43]. Hence, sTNF-R1 may be a useful, economical blood-based diagnostic tool for the early identification of subjects with CSVD before progression to dementia.

Besides neuroimaging markers, the second main aim of the study was to investigate sTNF-R1’s associations with cognitive decline. Although we did not find significant differences in the baseline *z*-scores among sTNF-R1 tertiles (Supplementary Figure S2), longitudinal analyses showed that CIND subjects with a higher sTNF-R1 declined more slowly in terms of global cognition than those at the lowest sTNF-R1 tertile. These results complement a recent study that reported elevated levels of CSF markers, including sTNF-R1, associated with decreased cognitive decline and a risk of conversion to AD in MCI subjects [44]. Since sTNF-R1 acts as a decoy receptor to inhibit TNF-induced signaling [13], it is possible that predementia subjects with higher sTNF-R1 levels may be protected from cognitive decline resulting from TNF-mediated neuroinflammatory or neurovascular injuries, as higher CSF levels of TNF were found to be associated with conversion to dementia [45]. Taken together, the present findings of higher sTNF-R1 being associated with more severe baseline lacunes but also a slower cognitive decline over the follow-up period, suggest a complex picture where robust responses to more severe CSVD-induced neuroinflammation resulted in better protection against cognitive decline in the longer term. Interestingly, the protective effects of sTNF-R1 may be specific to VCI, as sTNF-R1 changes were unrelated to the characteristic brain perturbations of AD (using MTA as the neuroimaging marker).

This study has several limitations. Firstly, our results on memory clinic patients might not be generalizable to the population at-large as there may be non-memory clinic patients with risk factor profiles that are not considered in our analyses. Secondly, as associations between baseline serum sTNF-R1 and CSVD neuroimaging markers were cross-sectionally analyzed, investigations into the temporal relationship between sTNF-R1 and the progression of the markers are needed. Thirdly, we were not able to detect any sTNF-R1 associations with clinical conversion (NCI to CIND or CIND to dementia) or with cognitive trajectories within the NCI group, due to the small sample size and relatively short follow-up period. Furthermore, whilst our finding of higher sTNF-R1 levels correlating with lacunes supports sTNF-R1 elevation as a putatively protective response to TNF-associated CSVD progression, we were not able to detect TNF directly due to platform and sample factors (data not shown), and correlations between blood TNF and sTNF-R1 levels are needed in future investigations to validate the functions of TNF-mediated inflammation and responses in VCI and dementia. Lastly, the relatively small sample size of the current study necessitates follow-up research with larger cohorts and a longer follow-up duration to more adequately study sTNF-R1 effects on cognitive trajectories stratified by CVSD severity.

## 5. Conclusions

To the best of our knowledge, this study is the first to examine serum sTNF-R1 associations with both neuroimaging CSVD markers, as well as cognitive trajectories in predementia subjects. Since vascular risk factors may influence the onset and progression of dementia [42], our multiple regression models controlled for potential confounding effects of vascular risk factors, and still found that the higher levels of serum sTNF-R1 in CIND subjects were associated with more severe lacunar infarcts but also with slower cognitive decline, suggesting a complex interaction between sTNF-R1 and CSVD-associated processes. Our study proposes sTNF-R1 as a prognostic peripheral biomarker for identifying patients with concomitant cerebrovascular pathologies, as well as for early identification of individuals at risk for VCI. However, further clinical studies using larger cohorts with longitudinal design are needed to validate the clinical utility of sTNF-R1. 

## Figures and Tables

**Table 1:** Demographic characteristics of cases and controls at baseline.

Characteristics	NCI (*n* = 93)	CIND (*n* = 103)	*p*-Value
Age, mean (SD)	68.9 (6.4)	74.1 (6.9)	<0.001 *
Female, *n* (%)	54 (58.1)	49 (47.6)	0.142
Years of education, mean (SD)	9.7 (4.7)	7.9 (4.4)	0.006 *
APOE4 carrier, *n* (%)	22 (23.7)	27 (26.2)	0.680
Hypertension, *n* (%)	53 (57.0)	69 (67.0)	0.149
Diabetes mellitus, *n* (%)	20 (21.5)	33 (32.0)	0.097
Cardiovascular disease, *n* (%)	5 (5.4)	14 (13.6)	0.052
Hyperlipidemia, *n* (%)	66 (71.0)	80 (77.7)	0.282
sTNF-R1, median (IQR), pg/mL	1126.5 (381.3)	1236.5 (587.4)	<0.001 *

CIND = cognitive impairment, no dementia; IQR = interquartile range; NCI = no cognitive impairment; *n* = number of cases; SD = standard deviation; sTNF-R1 = soluble tumor necrosis factor-receptor 1. * *p* < 0.05; Student’s *t*-test or Mann–Whitney U-test.

**Table 2:** Association between serum sTNF-R1 tertiles and CSVD markers at baseline ^1^.

sTNF-R1 (Tertiles)	WMH (ARWMC) MD (95% CI)	Number of Lacunes RR (95% CI)	Number of CMBs RR (95% CI)
*Model I*			
Lowest	0	1	1
Middle	0.34 (−0.90, 1.59)	1.68 (0.79, 3.58)	1.59 (1.00, 2.53)
	*p* = 0.590	*p* = 0.182	*p* = 0.051
Highest	1.43 (0.20, 2.65)	4.16 (2.15, 8.04)	2.02 (1.29, 3.16)
	*p* = 0.022 *	*p* < 0.001 *	*p* = 0.002 *
*Model II*			
Lowest	0	1	1
Middle	−0.21 (−1.43, 1.02)	2.08 (0.95, 4.56)	1.56 (0.91, 2.68)
	*p* = 0.738	*p* = 0.068	*p* = 0.110
Highest	0.18 (−1.22, 1.58)	6.91 (3.19, 14.96)	1.73 (0.97, 3.08)
	*p* = 0.800	*p* < 0.001 *	*p* = 0.061

ARWMC = age-related white matter changes; CSVD = cerebral small vessel disease; CI = confidence interval; CMBs = cerebral microbleeds; WMH = white matter hyperintensities; MD = mean difference; RR = rate ratio. CSVD markers were available for 186 subjects. * *p* < 0.05 using linear, Poisson or negative binomial regression. *Model I*: unadjusted; *Model II*: adjusted for age, gender, APOE4 carrier status, hypertension, diabetes mellitus, cardiovascular disease and hyperlipidemia. ^1^ Interpretation: the lowest sTNF-R1 tertile was set as a reference group. Linear regression models were used for WMHs by ARWMC scores: when MD > 0, subjects with the middle/highest sTNF-R1 tertile will have MD units higher (lower when MD < 0) in the ARWMC scores compared with those with the lowest sTNF-R1 tertile. Poisson and negative binomial regression models were used for lacunes and CMBs: subjects with the middle/highest sTNF-R1 tertile were RR times more likely to have as many lacunes or CMBs compared with those with the lowest sTNF-R1 tertile.

## Data Availability

Anonymized data derived from this study may be provided by the corresponding author upon reasonable request.

## Supplementary Materials

The following supporting information can be downloaded at: https://www.mdpi.com/article/10.3390/biom13030525/s1, Table S1: Summary of neuropsychological battery and component tests [22,46,47,48,49,50,51]; Table S2: Association between serum sTNF-R1 tertiles and MTA at baseline; Figure S1: Selection of study participants; Figure S2: Baseline global and specific cognitive domains z-scores of CIND subjects stratified by sTNF-R1 tertiles.
