# Supplementary material for: Elevated Soluble TNF-Receptor 1 in the Serum of Predementia Subjects with Cerebral Small Vessel Disease
Source: Biomolecules. 2023 Mar 13;13(3):525. doi: 10.3390/biom13030525 (PMC10046240; doi:10.3390/biom13030525)
Supplement: Supplementary file 1 [file biomolecules-13-00525-s001.zip › biomolecules-2236838-supplementary.pdf]

Salai *et al.* Elevated Soluble TNF-Receptor 1 in the Serum of Predementia Subjects with Cerebral Small Vessel Disease

Supplementary Figures and Tables

Table S1 Summary of Neuropsychological Battery and Component Tests

Cognitive Domain	Component Test(s)
1. Executive Function:	Verbal Fluency [1], Color Trail Test A&B [2]
2. Working Memory:	Digit Span Forward and Backward [3]
3. Language:	15-item Modified Boston Naming Test [4]
4. Visuospatial Function:	Rey Complex Figure Test-copy [5]
5. Visuomotor Speed:	Symbol Digit Modalities Test [6]
6. Memory:	Rey Complex Figure Test-immediate/delay recall and recognition [5], Hopkins Verbal Learning Test-immediate/delay recall and recognition [7]

Table S2 Association between serum sTNF-R1 tertiles and MTA at baseline

sTNF-R1	Significant MTA (n=49)<br>OR (95% CI)<br>*p*-value
**Model I**	
**Lowest**	1
**Middle**	**2.61 (1.02, 6.65)**
	***p* = 0.045**
**Highest**	**4.61 (1.88, 11.29)**
	***p* = 0.001**
**Model II**	
**Lowest**	1
**Middle**	2.19 (0.80, 6.00)
	p = 0.127
**Highest**	1.42 (0.49, 4.09)
	p = 0.518

Abbreviation: CI = confidence interval; MTA = Medial Temporal Lobe Atrophy; OR = Odds ratios.

MTA imaging were available for 186 subjects. **Bold values** indicate $p<0.05$ using binary logistic regression.

Model I: unadjusted; Model II: adjusted for age, gender, and cardiovascular disease.

Interpretation: Lowest sTNF-R1 tertile was set as a reference group. Binary logistic regression models to significant MTA: subjects with the middle/highest sTNF-R1 tertile were OR times more likely to have significant MTA compared to those with the lowest sTNF-R1 tertile.

Figure S1 Selection of participants

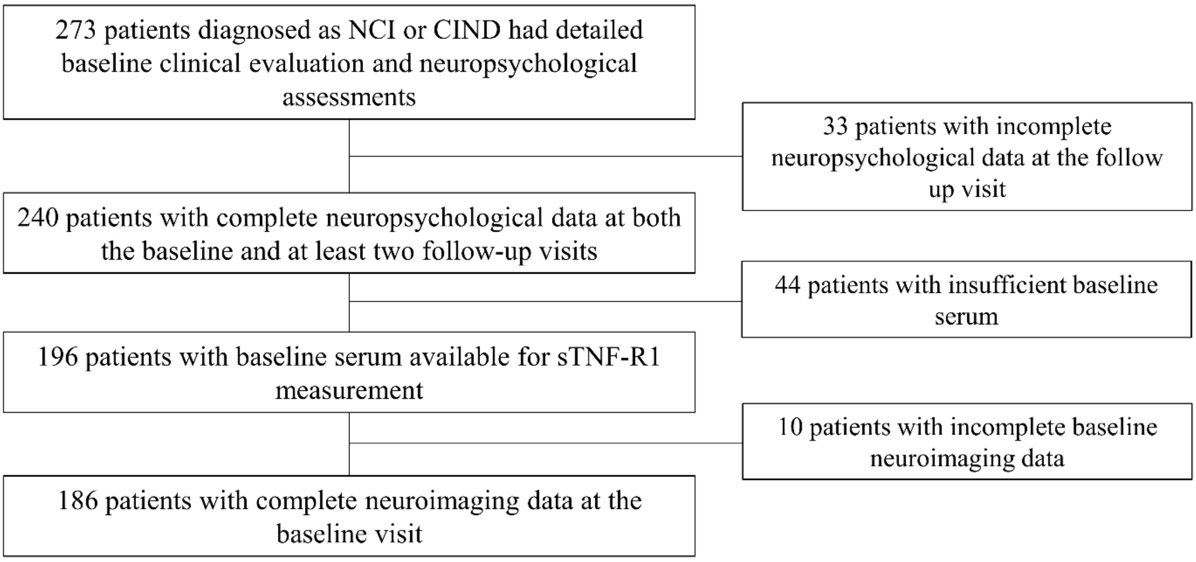

CIND = cognitive impairment no dementia; NCI = no cognitive impairment.

Figure S2 Baseline global and specific cognitive domains z-scores of CIND subjects stratified by the tertiles of sTNF-R1.

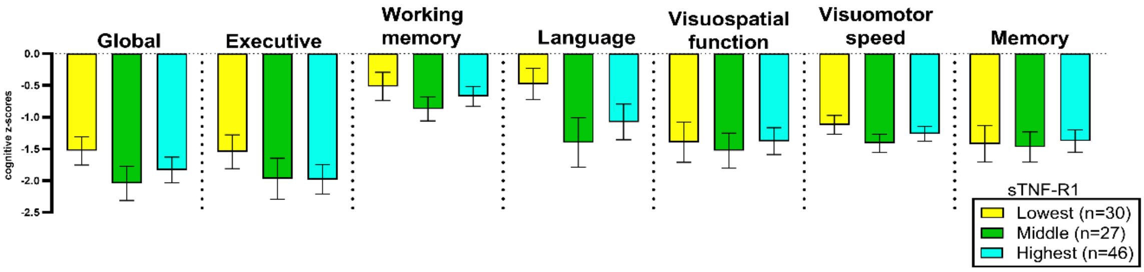

Bars are expressed as mean ± SEM. One-way analyses of variance (ANOVA), followed by *post-hoc* Bonferroni tests, was used to analyse the differences amongst tertiles of sTNF-R1 within each cognitive domain. n = number of cases.

REFERENCES

1. McKhann, G., et al., *Clinical diagnosis of Alzheimer's disease: Report of the NINCDS-ADRDA Work Group* under the auspices of Department of Health and Human Services Task Force on Alzheimer's Disease.* Neurology, 1984. **34**(7): p. 939-939.
2. D'Elia, L., et al., *Color Trails Test. Odessa, FL: Psychological Assessment Resources.* Inc Google Scholar, 1996.
3. Wechsler, D., *Subtest Administration and Scoring. WAIS–IV: Administration and Scoring Manual.* San Antonio, TX: The Psychological Corporation, 2009: p. 87-93.
4. Mack, W.J., et al., *Boston Naming Test: shortened versions for use in Alzheimer's disease.* Journal of gerontology, 1992. **47**(3): p. P154-P158.
5. Meyers, J., *Meyers scoring system for the Rey complex figure test and the recognition trial.* Psychological Assessment Resources, Odessa, FL, 1994.
6. Smith, A., *Symbol digit modalities test*. 1973: Western psychological services Los Angeles.
7. Brandt, J., *The Hopkins Verbal Learning Test: Development of a new memory test with six equivalent forms.* The clinical neuropsychologist, 1991. **5**(2): p. 125-142.
